# PRRSV Fetal Infection in Pigs as a Risk Factor for the Emergence of More Efficient Zoonotic Hosts for Swine-Origin Influenza Virus

**DOI:** 10.3390/vetsci13090983

**Published:** 2026-09-17

**Authors:** Uladzimir Karniychuk

**Affiliations:** 1 Department of Veterinary Biosciences, College of Veterinary Medicine, The Ohio State University, Columbus, OH 43210, USA; karniychuk.1@osu.edu; Tel.: +1-740-803-4364; 2 Vaccinology and Immunotherapeutics Program, School of Public Health, University of Saskatchewan, Saskatoon, SK S7N 5A2, Canada; 3 Veterinary Microbiology Department, Western College of Veterinary Medicine, University of Saskatchewan, Saskatoon, SK S7N 5A2, Canada; 4 Center for RNA Biology, The Ohio State University, Columbus, OH 43210, USA; 5 Infectious Disease Institute, The Ohio State University, Columbus, OH 43210, USA

**Keywords:** *Betaarterivirus*, PRRSV, influenza, zoonotic, spillover, one health, fetus, pig

## Abstract

Domestic pigs are susceptible to avian, swine, and human influenza A viruses (IAVs). This facilitates co-infections of different IAVs that result in genetic reassortment. In combination with husbandry practices such as high-density farming and frequent human–pig interactions, pigs serve as efficient zoonotic hosts for IAV. Despite significance in zoonotic transmission, factors influencing IAV pathogenesis, evolution, and transmission in pigs remain poorly studied. One factor that may alter IAV dynamics in pigs is co-infection with endemic swine viruses such as porcine reproductive and respiratory syndrome virus (PRRSV; *Betaarterivirus*; *Nidovirales*) that causes widespread disease in pigs on farms globally. During initial outbreaks, PRRSV triggers transplacental infections and massive fetal loss in naïve herds. Once herd immunity or vaccination is established, PRRSV may persist in affected herds, resulting in transplacental infections and the birth of piglets that appear normal or weak. These piglets are concerning from a zoonotic perspective because they may be immunocompromised and potentially serve as more efficient hosts for IAV, characterized by prolonged IAV shedding, enhanced viral replication, impaired seroconversion, and consequently extended co-infection windows that favor reassortment. Here, I summarize studies supporting the hypothesis that fetal exposure to PRRSV can alter IAV pathogenesis in surviving piglets, potentially enhancing IAV pathogenesis and transmission. I also highlight key experiments that need to be conducted to validate or invalidate a causal relationship between fetal PRRSV infection, persistent immunopathology in surviving piglets, and the resulting permissive environment for enhanced IAV evolution and transmission. Addressing this knowledge gap is essential for advancing our understanding of PRRSV and IAV biology in its zoonotic pig host and for developing effective strategies to mitigate the risk of IAV spillovers to humans.

## Simple Summary

Domestic pigs are susceptible to avian, swine, and human influenza A viruses (IAVs), facilitating co-infections that can generate new virus variants. Combined with high-density farming and frequent human–pig interactions, this makes pigs important hosts for IAV emergence and transmission to humans. One factor that may alter IAV infection in pigs is porcine reproductive and respiratory syndrome virus (PRRSV), which causes widespread disease on farms globally. PRRSV can persist in affected herds, resulting in fetal infections and the birth of piglets that appear healthy or weak. These piglets may have impaired immune responses and potentially serve as more efficient hosts for IAV, with increased viral replication, prolonged shedding, more severe disease, and greater opportunities for transmission. Here, I summarize evidence supporting the hypothesis that fetal PRRSV exposure alters IAV infection in surviving piglets and highlight key experiments needed to test this hypothesis. Addressing this knowledge gap may help develop strategies to reduce IAV spillovers from pigs to humans.

## 1. Introduction

Domestic pigs (*Sus scrofa domesticus*) express avian-like (SA-α2,3) and human-like (SA-α2,6) sialic acid receptors, making them uniquely susceptible to avian, human, and swine influenza A viruses (IAVs). This dual receptor expression facilitates coinfections that result in genetic reassortment and the emergence of novel IAV strains [1]. The mortality rate in IAV-infected pigs is low, and in combination with husbandry practices such as high-density farming and frequent human–pig interactions, pigs serve as efficient zoonotic hosts for IAV. Vaccination of pigs mitigates IAV transmission within herds and to humans. However, while modified live IAV vaccines stimulate both humoral and cell-mediated immunity, they have several practical limitations [2,3,4,5,6,7,8]. Existing inactivated and subunit IAV vaccines only provide protection against homologous strains of viruses and not against genetic variants that constantly emerge in the field [9,10]. The 2009 H1N1 influenza pandemic resulted from a reassortment of swine, avian, and human influenza viruses within pigs. Between April 2009 and April 2010, the US population experienced approximately 12,469 deaths and 274,304 hospitalizations. The economic impact in the US was around $2 billion in lost labor productivity. The pandemic resulted in an estimated loss of 28,126 quality-adjusted life years, with 40% attributed to mortality [11,12].

Beyond the 2009 H1N1 influenza pandemic, several significant pig-to-human IAV spillovers have occurred globally and in the US (discussed below), leading to human deaths, changes in public health policy, and economic impact. In addition to the zoonotic risks associated with human–pig interactions on industrial swine farms, agricultural fairs and exhibitions have emerged as critical settings for the amplification of swine influenza viruses and spillover into the broader human population. This extends beyond occupational exposure in pork production and is exemplified by research conducted at US national agricultural fairs [13,14,15,16,17,18,19,20,21,22,23,24,25,26,27,28,29,30,31,32,33,34,35,36].

One factor that may significantly alter IAV dynamics in pigs is co-infection with endemic swine viruses such as porcine reproductive and respiratory syndrome virus (PRRSV; *Betaarterivirus*; *Nidovirales*), a pathogen that causes widespread reproductive and respiratory disease in pigs on farms globally.

PRRSV was first identified in the late 1980s and early 1990s, independently in Europe and North America, following outbreaks of severe reproductive failures and respiratory illness. Since its discovery, PRRSV rapidly disseminated worldwide, now representing a persistent threat to global pig production [37].

PRRSV primarily transmits among pigs through direct contact and vertical transmission. Additionally, PRRSV spreads indirectly via contaminated fomites [38]. Aerosol transmission has also been documented, particularly under high-density swine farm conditions, allowing airborne spread between farms located several kilometers apart [39]. The stability of PRRSV in the environment, coupled with the frequent movement of animals and farm workers, contributes significantly to its rapid dissemination.

Clinically, PRRSV infection in pregnant pigs results in severe reproductive losses, including increased rates of late-term abortion, early farrowing, stillbirth, mummified fetuses, and weak-born piglets [40]. The virus crosses the placenta, establishing persistent infections in fetal tissues. In young piglets and growing pigs, PRRSV causes respiratory disease and increased susceptibility to secondary infections, reduced growth rates, and elevated mortality [41,42,43,44,45]. The economic burden imposed by PRRSV is substantial, making it one of the most economically significant diseases in swine production. In the US alone, annual economic losses attributed to PRRSV exceed $1.2 billion [46]. Globally, PRRSV imposes severe economic damage, particularly in high-density pork-producing regions such as Europe and Asia [46].

Eradicating PRRSV requires complete herd replacement, as vaccination is largely ineffective due to the PRRSV’s exceptionally high mutation rate (4.71–9.8 × 10^−2^ substitutions/site/year) [47]—enabling rapid escape from vaccine-induced immunity [48,49]. We also previously demonstrated that vaccine-induced immunity enhances PRRSV infection in the endometrium of pregnant pigs and fails to prevent transplacental transmission and fetal infections [50]. Thus, many industrial swine farms in the US and globally operate under an endemic PRRSV infection status due to the costs of animal replacement and ease of viral reintroduction [51,52]. This endemicity allows the virus to persist, including through clinical and subclinical transplacental and fetal infections.

Surveillance in the US, Europe, and Asia confirms widespread PRRSV and IAV co-infections, particularly in intensive swine production systems, amplifying disease severity and economic losses in pig production [53,54]. Field epidemiological reports describe an increased incidence and severity of secondary bacterial infections, e.g., *Streptococcus suis*, *Haemophilus parasuis*, *Mycoplasma hyopneumonia*, *Actinobaccillus pleuropneumonia*, *Salmonella* spp. and swine influenza virus, in pig herds following PRRSV invasion [41,42,43,44,45].

Fetal distress via maternal malnutrition [55,56], stress [57,58], or maternal and fetal infections [59,60,61], can affect long-term health and immune responses in mouse and human offspring. During initial outbreaks, PRRSV triggers transplacental infections and massive fetal loss in naïve herds. Once herd immunity or vaccination is established, PRRSV persists through subclinical transplacental infections, resulting in the birth of piglets that appear normal or weak. These piglets are concerning from a zoonotic perspective, as they remain in production but are immunocompromised, potentially serving as more efficient hosts for other pathogens. For example, in utero-acquired impairment of mucosal and pulmonary macrophage responses might enhance IAV replication in the upper respiratory tract and lungs, leading to prolonged viral shedding. Another possibility is that in utero-acquired impairment of adaptive humoral and cellular immune responses may extend co-infection windows, thereby increasing opportunities for IAV reassortment.

Our and others’ research has provided substantial data supporting immunocompromised states in piglets infected with PRRSV during fetal life (discussed below). However, despite more than 35 years of research since the emergence of PRRSV and decades of work on IAV in pigs, the potentially critical PRRSV–pregnant pig–fetus–offspring–IAV interactions have not been proposed or experimentally studied. This knowledge is critical for advancing the basic understanding of IAV biology and developing strategies to mitigate IAV emergence in swine herds and spillovers to humans.

## 2. Historical Data of Overlapping Emergence of PRRSV and Swine-Origin IAV Spillovers

There is no continuous public time series consolidating PRRSV and swine IAV incidence data or spillovers. Swine IAV monitoring, as well as PRRSV monitoring, is usually voluntary and incomplete. Most available information is derived from isolated surveys or outbreak reports of specific viruses [62]. This limitation precludes the application of statistical methods—such as ARIMA time-series models or Granger causality tests—to annual PRRSV prevalence and IAV case counts. Thus, only proxy indicators are summarized below (Table 1), suggesting that PRRSV emergence could facilitate IAV transmission and spread on swine farms.

Retrospective studies found no PRRSV antibodies (Abs) in the US and Europe before 1980, but by 1985–1986, seropositive herds were retrospectively identified [63]. Classical swine H1N1 viruses persisted and remained antigenically stable from 1918 until the 1980s [64]. Following the clinical emergence of PRRSV in 1987, dozens of sporadic human infections with divergent swine IAVs were documented in the 1990s and 2000s, far more than in prior decades [65,66]. These dates place PRRSV’s emergence roughly concurrent with some early swine-origin IAV spillover events.

**Table 1:** Key historical milestones in the emergence and spread of PRRSV and swine-origin IAV.

Geographical Region	PRRSV Events	IAV Events
North America	1979, earliest PRRSV Ab evidence (retrospective) [63], Canada	1976, swine flu outbreak in soldiers (H1N1) [67], USA (New Jersey)
	1985, first seropositive pigs identified [63], USA (Iowa) 1986, PRRSV seropositive pigs detected [63], USA (Minnesota) 1987, first recognized PRRS outbreaks (“Mystery Swine Disease”) [68], USA	1988, human fatality from swine H1N1 [69], “Wisconsin Fair” case, USA
	1991, the causative virus isolated in the USA (PRRSV strain VR-2332) [70]	1998, triple-reassortant H3N2 virus emerges in pigs [71,72], USA (Midwest) 2005–2007, sporadic swine-to-human transmission cases in the USA [65]
Asia	1985, Retrospective serologic evidence of PRRSV in South Korea [73,74] 1988, First Asian outbreaks [75], Japan	Early 2000s, diverse influenza strains (H3N2, H9N2) co-circulate in pigs [65], China (Guangdong and other provinces)
	2006–2007, highly pathogenic PRRSV (HP-PRRS) outbreak with 50–100% morbidity and up to 20–100% mortality, China, Vietnam, Thailand, Cambodia	2016–2020, emergence of G4 EA H1N1 in pigs [76], China
Europe	1988–1990, pigs tested seropositive in 1988–1989, and a major outbreak occurred in 1990 [63], Germany 1991, the causative virus isolated in the Netherlands (Lelystad virus) [77] 2007, East Europe, a highly pathogenic East European subtype 3 was isolated and characterized [78]	Early 1990s, Avian-like and reassortant viruses emerged in European pigs (H1N1, H3N2) [65]

In the US, the first unexplained “mystery swine disease” (later identified as PRRS) was noted in 1987 [37]. Remarkably, within a year, in 1988, swine IAV spilled to humans and caused a fatal infection [79]. This tragic case, occurring as PRRSV was quietly spreading in pig herds, underscored pigs’ role as a source of zoonotic flu and raised concern that new swine viruses could reach humans. PRRSV became endemic in US pig farms by the early 1990s [37], and soon after, the diversity of swine influenza expanded. In 1998, a novel triple-reassortant H3N2 influenza virus (with gene segments from human, avian, and swine flu) was detected in US pigs [64,67,68,80], demonstrating the striking temporal overlap with a period when PRRSV became widespread. It marked the first major deviation from the classic swine H1N1 lineage and rapidly spread through North American herds. Variants of this 1998 virus subsequently gave rise to new swine H1N2 and H1N1 strains, and notably six of the eight gene segments of the 2009 pandemic H1N1 virus came from descendants of these 1998 swine strains.

In Asia, PRRSV was first reported in Japan in 1988 and subsequently became widespread across East Asia in the 1990s. China’s huge pig industry has become a hotspot for influenza mixing. By the 2000s, avian H9N2 and human H3N2 viruses were co-circulating in Chinese pigs, resulting in the emergence of new reassortants [65].

Europe experienced similar events. PRRSV was first recognized in 1990 in Germany [37]. In the early 1990s, European pigs were found to harbor novel avian–human reassortant flu viruses. For example, an H3N2 containing avian internal genes and human-adapted surface proteins arose in European swine and infected at least two children in the Netherlands in 1993 [65].

The above proxy analysis does not account for major changes in swine production systems and diagnostic capabilities that occurred during the same period. During the late 20th and early 21st centuries, swine production increasingly shifted toward larger, high-density, highly interconnected production systems, with frequent movement of pigs between farms and production stages [81]. Such conditions can independently promote sustained IAV circulation, transmission between herds, introduction of genetically distinct viruses, and opportunities for viral reassortment and evolution. Larger herd size and high pig density have been associated with increased IAV infection risk, while high turnover and continuous introduction of susceptible piglets can facilitate long-term viral persistence within production systems. In parallel, improvements in molecular diagnostics, sequencing, and active surveillance substantially increased the ability to detect IAV infections, reassortants, and zoonotic transmission events that may previously have gone unrecognized. Therefore, the apparent increase in IAV diversity and documented spillover events following PRRSV emergence cannot be attributed to PRRSV alone and likely reflects multiple interacting biological, production, and surveillance factors. Nevertheless, PRRSV-associated immunopathology may represent one additional biological factor contributing to an increasingly permissive environment for IAV circulation, persistence, and evolution in intensively managed swine populations. Further studies using systematic, continuous time-series data integrating real-time PRRSV and swine IAV incidence and zoonotic spillover events, followed by appropriate statistical analyses, are needed to determine potential associations between PRRSV prevalence and IAV incidence.

## 3. Recent On-Farm Studies Supporting the Role of PRRSV in Exacerbating IAV Infection in Pigs

A 2024 Spanish study is likely the only comprehensive investigation to directly examine swine IAV dynamics before and after the introduction of a new PRRSV strain on two commercial pig farms [45]. Following the incursion of a virulent PRRSV strain, the IAV infection rate in piglets increased dramatically from 20–50% to 70%. Moreover, a significant proportion of influenza-positive pigs began shedding IAV for ≥1 week, a duration rarely observed prior to the PRRSV outbreak. This unusually prolonged IAV shedding was strongly associated with PRRSV-positive status. Importantly, PRRSV-infected piglets often fail to mount a detectable Ab response to IAV and can be reinfected during the nursery period (3–8 weeks of age). Among pigs that tested positive for IAV only once, 10.5% were co-infected with PRRSV. In contrast, among those with repeated IAV detection, 36.6% were PRRSV-positive at the time of their first IAV infection, and 50% of pigs with two or more consecutive IAV-positive samplings were co-infected with PRRSV [45].

In this study, piglets were followed from birth to 8–12 weeks of age and sampled longitudinally at 1, 3, and 4–8 weeks post-birth [45]. Following the introduction of a highly virulent PRRSV strain belonging to a newly emerging clade, the virus was detected in up to 30% of newborn piglets as early as one week of age. Although it is not possible to definitively determine whether these one-week-old piglets acquired PRRSV before or after birth, transplacental transmission during fetal life is a plausible explanation. This route is supported by the fact that adult pigs on these farms were routinely vaccinated, yet it is well established that commercial live vaccines do not provide full protection against respiratory or transplacental infection with heterologous PRRSV strains, particularly highly pathogenic variants [82,83,84,85,86,87,88]. Moreover, inactivated PRRSV vaccines are ineffective at protecting pregnant sows against transplacental and fetal infections, even after multiple booster vaccinations. For example, we previously demonstrated that immunity induced by inactivated vaccines can enhance PRRSV replication in the endometrium of pregnant pigs and fails to prevent transplacental transmission and fetal infection, even after three booster doses and homologous challenge with a moderately pathogenic PRRSV strain [50].

Another observation in this study [45] supporting the hypothesis of fetal-origin PRRSV infection was the unusually prolonged duration of PRRSV nasal shedding—averaging approximately one month—which exceeds what is typically reported for many PRRSV strains. Notably, such extended virus persistence has previously been reported in immunocompromised piglets which acquired PRRSV during fetal life after experimental maternal/transplacental infection [41].

Future field studies on farms with PRRSV and IAV co-circulation should be designed to clearly distinguish between two potential routes of PRRSV infection—acquired in utero versus acquired postnatally—and determine their independent effects on subsequent IAV infection.

## 4. Experimental PRRSV and IAV Coinfection Studies in Piglets

Several studies have reported conflicting results regarding the pathogenesis of disease in piglets co-infected with PRRSV and IAV after birth (with no fetal PRRSV infection). One study found that sequential infection with PRRSV followed by IAV three days later led to more severe clinical disease and growth retardation compared to IAV infection alone, while only modestly affecting IAV shedding [89]. In contrast, another study reported that simultaneous co-infection with PRRSV and swine IAV did not significantly alter clinical signs, lung lesions, immune responses, or viral replication in the respiratory tract compared to IAV infection alone [90]. More recently, it was shown that simultaneous co-infection did not impair the development of immune responses relative to single IAV infections [91].

In all three studies [89,90,91], healthy young piglets were co-infected with PRRSV and IAV either simultaneously or three days apart. A key limitation of this experimental design is that it does not capture the systemic or lung-specific immunopathological sequelae that PRRSV may induce beyond the acute phase. Supporting this hypothesis, a study in which piglets were infected with IAV three days after initial PRRSV infection reported more severe clinical disease [89]. In contrast, studies using simultaneous PRRSV and IAV inoculation did not observe such disease exacerbation [90,91]. Porcine reproductive and respiratory syndrome virus can persist in the lungs and cause lesions for up to 3–4 weeks [78]. Therefore, to determine whether PRRSV-induced lung pathology and immunopathology exacerbate IAV infection in young piglets, studies involving primary PRRSV infection followed by IAV co-infection at extended intervals, 1–4 weeks later, are warranted. These future experiments at different time points must be conducted under standardized experimental conditions. Three different groups performed the three previous co-infection studies in postnatal piglets [89,90,91], potentially introducing confounding factors such as differences in virus strains and doses, animal age and sex, and housing conditions.

## 5. PRRSV-Induced Immunopathology in Fetuses and Persistent Immunopathology Sequelae in Affected Piglets

**Figure 1:**
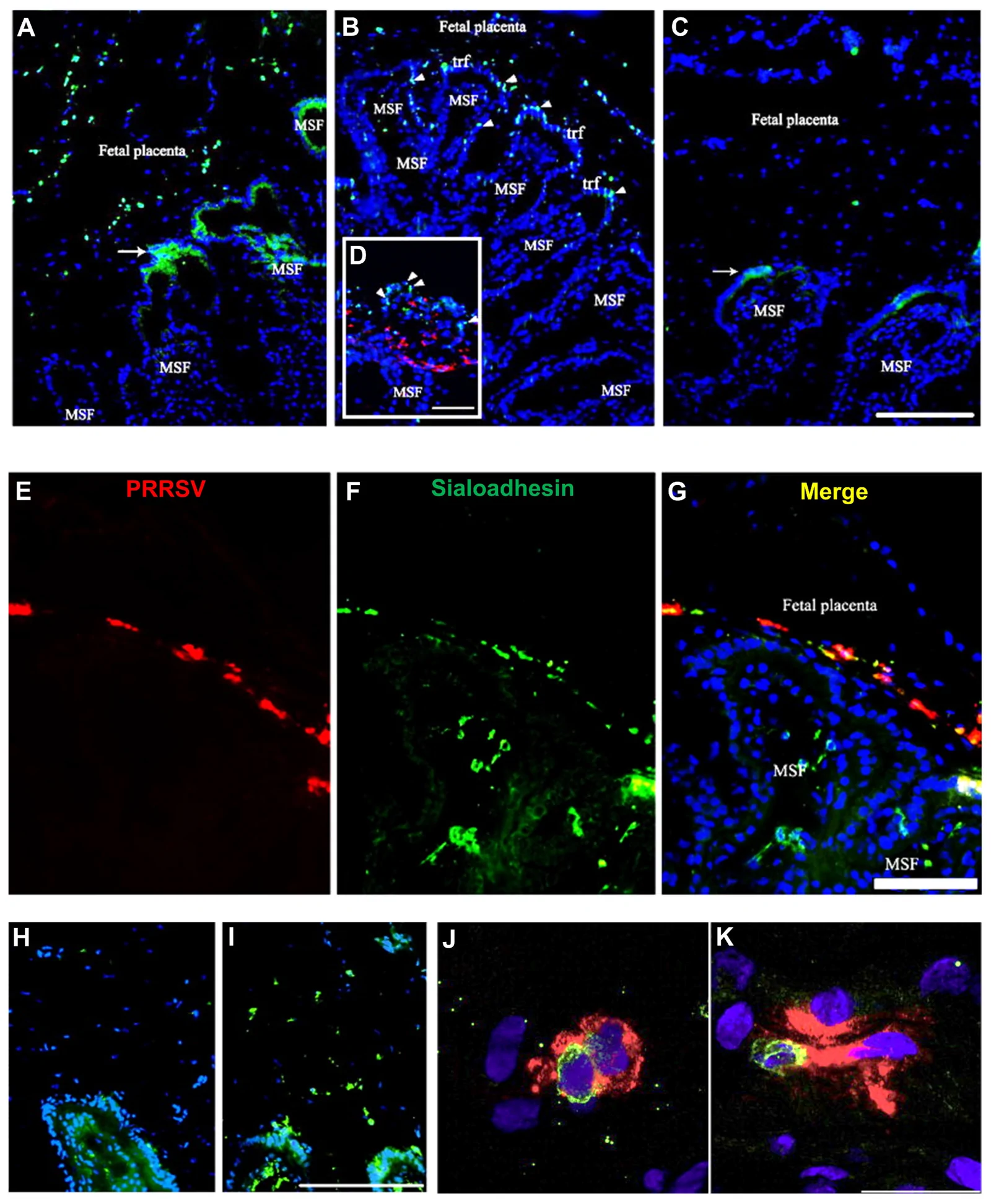
Complex PRRSV–host immune cell interactions and pathology during PRRSV infection in the fetal placenta. (**A**–**C**) Staining for apoptosis (green) and nuclei (blue) in the fetal implantation sites of the uterus derived from sows inoculated with PRRSV at 90 days of gestation and sampled at 10 days after maternal PRRSV inoculation (**A**,**B**) and healthy sow (**C**) [97]. PRRSV infection in the fetal placenta is associated with the significant increase in apoptotic cells (**A**–**C**) [97]. MSF: maternal secondary fold. Most apoptotic cells were in the fetal placental mesenchyme (**A**). In a few sections, apoptotic cells (arrowhead) were spaced within the trophoblast (trf) layer (**B**,**D**). Staining for apoptosis (green), CD163 (red) and nuclei (blue) revealed that these cells are not CD163-positive macrophages (**D**) [97]. Bar, 200 μm (**A**–**C**) and 100 μm (**D**). Intense autofluorescence of the trophoblasts is arrowed (**A**,**C**). (**E**–**G**) PRRSV has tropism to sialoadhesin-positive macrophages in the fetal placenta [97]. Double immunofluorescence staining for PRRSV (red), sialoadhesin (green), and nuclei (blue) in the fetal implantation sites of the uterus derived from sows inoculated with PRRSV at 90 days of gestation and sampled at 10 days post-inoculation [97]. All PRRSV-positive cells in the endometrium and fetal placental mesenchyme were sialoadhesin-positive [97]. Bar, 100 μm. (**H**,**I**) Immunofluorescence staining of sialoadhesin (green) and Hoechst staining of nuclei (blue) in the fetal placenta derived from PRRSV-inoculated (**I**) and non-inoculated control sows (**H**) [98]. Bar, 200 μm. PRRSV infection was associated with an increased number of sialoadhesin-positive cells in the fetal placenta (**I**) [98]. (**J**,**K**) Immunofluorescence staining for CD8 marker, sialoadhesin and PRRSV in the placenta derived from a PRRSV-inoculated sow [98]. Bar, 25 μm. (**J**) Placental CD8-positive cell (green) is interacting with sialoadhesin-positive cell (red); Hoechst staining of cell nuclei (blue). (**K**) Placental CD8-positive cell (green) is interacting with PRRSV-positive cells (red); Hoechst staining of cell nuclei (blue). PRRSV infection was associated with an increased number of sialoadhesin-positive and CD8-positive cells in the fetal placenta [98]. Images were made using the Leica TCS SPE-II laser scanning spectral confocal system (Leica Microsystems GmbH) linked to a Leica DM2500 microscope (Leica Microsystems GmbH). Figures are adapted from previous publications [97,98].

**Figure 2:**
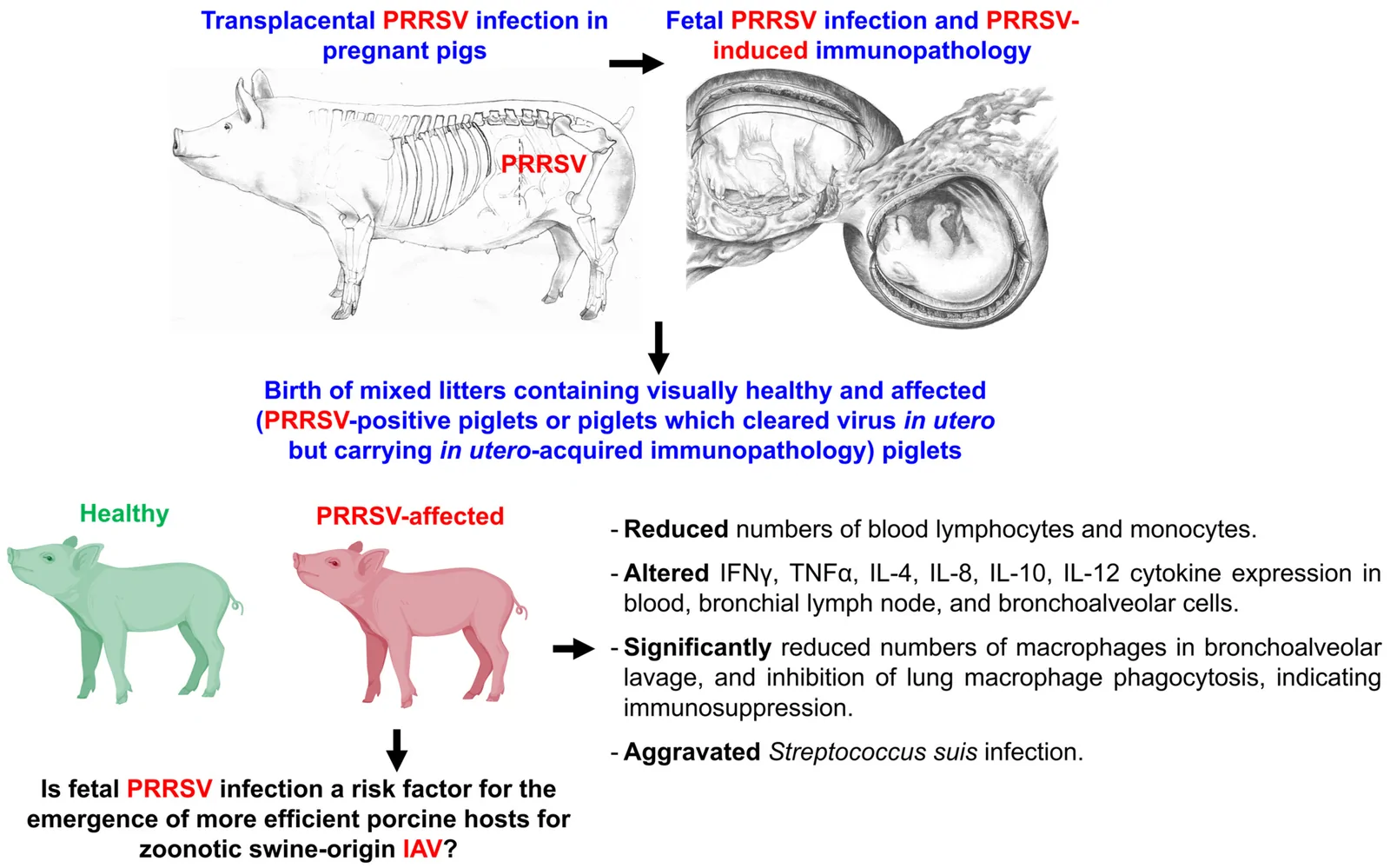
PRRSV-induced fetal immunopathology persists postnatally and is associated with increased susceptibility to bacterial infections in affected piglets. PRRSV-induced immunopathology acquired during fetal life may persist in surviving piglets after birth. In support, piglets exposed to PRRSV during fetal life had reduced numbers of blood lymphocytes and monocytes [103]. Exposed piglets also had altered IFN*γ*, TNFα, IL-4, IL-8, IL-10, IL-12 cytokine expression in blood, bronchial lymph node, and bronchoalveolar cells, suggesting initial activation with subsequent immune exhaustion and immunosuppression [104,105]. In another study, piglets that acquired PRRSV during fetal life and carried it after birth had significantly reduced (PRRSV-positive piglets—40, 77 and 76% versus Control—93% at 2, 4 and 6 weeks of age) numbers of macrophages in bronchoalveolar lavage, and inhibition of lung macrophage phagocytosis, indicating immunosuppression [41]. In experimental conditions, piglets affected with PRRSV during fetal life and bearing the virus after birth had aggravated infection caused by zoonotic *Streptococcus suis* bacteria [103], which naturally infects pigs and humans. It is unknown yet whether PRRSV-induced immunopathology acquired during fetal life may affect IAV pathogenesis, evolution, and transmission in immunocompromised piglets. Portion of figure (piglets) was created in BioRender. Karniychuk, U. (2026) https://BioRender.com/s8p5mj0.

In the above studies, healthy young piglets were co-infected with PRRSV and IAV [89,90,91]. However, fetal PRRSV infection—through its detrimental effects on developing immunity and induction of in utero immunopathology which persists after birth in piglets—may impair the host response to subsequent IAV infection and modify pathogenesis. Supporting this hypothesis, our research and research from other groups have demonstrated that PRRSV induces fetal immunopathology (Figure 1) that persists postnatally and is associated with increased susceptibility to bacterial infections in affected piglets (Figure 2).

PRRSV-infected pig fetuses show significantly increased levels of interferon alpha (IFNα) in blood serum, along with upregulated IFNα gene expression in the fetal spleen and thymus [92]. It is known that the increase in IFNα can lead to immune activation, inflammation, and immunopathology [93]. While type I IFNs are acknowledged for antiviral functions, increasing data suggest that excessive levels are toxic [94], e.g., increased IFNα levels in humans and primates are associated with immune activation and dysfunction [95]. We found that PRRSV has strong tropism for placental cells, leading to widespread apoptosis, inflammation, and a marked increase in sialoadhesin (Sn)-positive macrophages (Figure 1) [50,96,97,98,99]. Sialoadhesin is the inflammatory macrophage marker [100] activated by IFNα [101]. The increase in Sn expression was also confirmed by another group in the PRRSV-positive pig placenta and fetal thymus [102]. This and related immunopathology acquired during fetal life may persist in surviving piglets after birth (Figure 2). In support, piglets exposed to PRRSV during fetal life had reduced numbers of blood lymphocytes and monocytes [103]. Exposed piglets also had altered IFN*γ*, TNFα, IL-4, IL-8, IL-10, IL-12 cytokine expression in blood, bronchial lymph node, and bronchoalveolar cells, suggesting initial activation with subsequent immune exhaustion and immunosuppression [104,105]. In another study, piglets that acquired PRRSV during fetal life and carried it after birth had significantly reduced (PRRSV-positive piglets—40, 77 and 76% versus Control—93% at 2, 4 and 6 weeks of age) numbers of macrophages in bronchoalveolar lavage, and inhibition of lung macrophage phagocytosis, indicating immunosuppression [41]. PRRSV-positive piglets also had significantly increased frequency of blood and lung CD8^+^ T cells [41], which may reflect immune activation and subsequent exhaustion [106].

Under experimental conditions, piglets affected by PRRSV during fetal life and carrying the virus after birth showed dramatically increased susceptibility to the zoonotic bacterium *Streptococcus suis*, with mortality increasing from 6% or 22% in control PRRSV-infection-only or *Streptococcus suis*-infection-only piglet groups, respectively, to 91% in PRRSV-*Streptococcus suis* dual-infection group [103] (all piglets in the mock-infected group remained healthy). Although *Streptococcus suis* is a systemic bacterial pathogen and therefore differs fundamentally from respiratory IAV, this study provides proof-of-principle that fetal PRRSV infection can create a persistent postnatal host phenotype that profoundly alters susceptibility to a subsequent infection. Importantly, fetal PRRSV infection affects multiple components of innate and adaptive immunity that are also involved in antiviral defense, including macrophage populations and T-cell responses, and cytokine signaling. Thus, while the current experimental data does not prove that increased susceptibility to *Streptococcus suis* directly predicts increased susceptibility to IAV, these findings provide biological justification for testing whether PRRSV-induced postnatal immunopathology also alters the outcome of infection with an unrelated respiratory pathogen such as IAV. Unlike systemic *Streptococcus suis* infection, IAV is predominantly restricted to the respiratory tract; therefore, increased IAV replication localized in the respiratory tract or prolonged shedding could theoretically occur without increased mortality, potentially creating clinically surviving pigs with greater opportunities to transmit and maintain IAV. This possibility remains hypothetical and requires direct experimental testing.

## 6. Key Experiments to Define a Relationship Between Fetal PRRSV Infection, Persistent Immunopathology in Surviving Piglets, and Altered IAV Infection Phenotype

Only the key experiments are highlighted in Table 2, as many molecular, mechanistic, and functional studies could subsequently be designed if the PRRSV–IAV interaction hypothesis raised in this paper is confirmed. Discussion of molecular mechanistic studies would be premature until the fundamental effects of fetal PRRSV infection on IAV replication, associated clinical and histopathological outcomes, viral evolution, and transmission in offspring are established. The expected outcomes of the suggested experiments (Table 2) that would support the proposed hypothesis include enhanced IAV replication, exacerbated clinical signs and histopathological lesions, altered IAV evolution, and increased IAV transmission in PRRSV-affected piglets compared with controls.

## 7. Conclusions and Perspective

While established and regularly emerging highly pathogenic PRRSV strains cause widespread reproductive and respiratory disease in pigs globally, it remains unknown whether fetal exposure to PRRSV alters IAV pathogenesis, evolution, or transmission in affected piglets. Addressing this gap is critical for advancing fundamental knowledge of IAV biology and for improving strategies to mitigate zoonotic spillover risk. Extensive evidence from our research [49,50,78,96,97,98,99] and others [41,92,102,103,104,105] demonstrates that PRRSV affects fetal immune cells, leading to immunopathology that persists postnatally. These findings strongly support the need for future studies to directly test causal links between fetal PRRSV infection, postnatal immunopathology, and increased susceptibility to IAV replication and transmission in affected piglets. In addition, although this hypothesis paper focuses specifically on fetal PRRSV infection, other swine viruses which cause transplacental infections and are associated with immune dysregulation may warrant similar investigations. For example, future studies could examine whether porcine circoviruses [111], known to cause fetal infection in pigs, alter IAV infection, replication, or transmission in affected offspring.

## Figures and Tables

**Table 2:** Experimental roadmap to define a relationship between fetal PRRSV infection, persistent immunopathology in surviving piglets, and altered IAV infection phenotype.

Experimental Sequence	Experimental Procedures	Experimental Details
1	PRRSV infection of pregnant pigs	Intranasal infection of pregnant pigs negative for PRRSV antibodies. PRRSV causes transplacental and fetal infections in pregnant pigs only after maternal inoculation during early (20–25 days) or late (85–100 days) pregnancy [49,96] (the full term in pigs is 114–116 days). Infection at early pregnancy leads to fetal resorption and loss of pregnancy. In contrast, infection at 85–100 days of pregnancy leads to transplacental and fetal infections, causing, depending on the strain and dose, massive fetal death, the mix of stillborn and live-born piglets, or mostly surviving PRRSV-infected fetuses/piglets [41,103,104,105]. Mock-infected pregnant pigs should be included in the study.
2	Confirmation of in utero PRRSV exposure in live-born piglets	Successful PRRSV exposure of each newborn piglet is confirmed by testing (PCR and/or by infectious virus isolation and titration) individual amniotic membranes attached to the piglet’s skin at birth, the expelled placenta, and nasal secretions for PRRSV shedding. Piglets born to mock-infected pregnant pigs remain negative for PRRSV.
3	Confirmation of in utero-acquired immunopathology in live-born piglets	A range of classical and state-of-the-art immunological assays should be applied to quantitatively compare baseline humoral and cellular immune responses, as well as responses following IAV infection (described below). Longitudinal live-animal sampling can assess responses in blood plasma/serum, peripheral blood cells, and nasal mucosal samples. Dedicated studies can evaluate nasal mucosal and lung immune responses using tissues and cells collected postmortem. All immunological parameters should be compared between control and PRRSV-exposed groups.
4	IAV infection of control and PRRSV-affected piglets	Piglets from the control and PRRSV-exposed groups should be inoculated intranasally with IAV. Ideally, at least in initial studies, pregnant pigs and newborn piglets should be negative for IAV antibodies. Pregnant pigs should therefore be obtained from a high-health-status herd routinely monitored for IAV infection and antibodies. However, most adult pigs, at least in the US, have IAV antibodies due to vaccination or natural exposure. Because pigs have epitheliochorial placentation, maternal antibodies are not transferred to piglets in utero but are acquired after birth through colostrum. Thus, cesarean-derived, colostrum-deprived piglets can be used in these studies. Also, maternal colostrum-derived IAV antibodies typically wane within 4–8 weeks of piglet age, a period that coincides with the onset of clinical disease in piglets during some on-farm IAV outbreaks. If maintaining piglets until 8 weeks of age (~30 kg, 66 lb) is impractical because of facility or animal-care limitations, piglets can be inoculated at 4 weeks of age (~7 kg, 15 lb) using combined intranasal and intratracheal inoculation, a standard approach for studying IAV infection, transmission, and pathogenesis, thereby minimizing potential interference from residual maternal IAV antibodies [107,108,109,110].
5	IAV infection studies	The initial study should focus on defining the fundamental effects of fetal PRRSV infection on IAV replication (infectious titers and RNA loads in upper and lower respiratory tract tissues and nasal secretions), associated clinical and histopathological outcomes (standard IAV clinical readouts and lung histopathology), viral evolution (plaque purification or limiting-dilution cloning combined with IAV NGS), and transmission (co-housing IAV-infected PRRSV-affected or control piglets with healthy contact piglets to compare transmission rates) in PRRSV-affected versus healthy control piglets.

## Data Availability

The original contributions presented in this study are included in the article. Further inquiries can be directed to the corresponding author.
